# The revised version of the Physical Self-Description Questionnaire-Short for Chinese middle school students

**DOI:** 10.3389/fpsyg.2025.1513882

**Published:** 2025-06-05

**Authors:** Ruijin Cai, Yaru Guo, Chunyu Lu

**Affiliations:** ^1^Department of Physical Education, Shanghai Institute of Technology, Shanghai, China; ^2^Key Laboratory of Adolescent Health Assessment and Exercise Intervention, Ministry of Education, College of Physical Education and Health, East China Normal University, Shanghai, China; ^3^Department of Physical Education, Shibei Junior Middle School, Shanghai, China

**Keywords:** Chinese, middle school students, physical self-concept, PSDQ-S, revision

## Abstract

**Objective:**

Chinese version of the Physical Self-Description Questionnaire-Short (PSDQ-S) was revised, and its reliability, validity, and gender invariance were tested among Chinese middle school students.

**Methods:**

A convenience sampling method was used to select 1,632 middle school students in grades 7-12 (12-18 years old) in Shanghai for the PSDQ-S questionnaire survey, and SPSS 24.0 and Mplus 8.3 software were used for data analysis.

**Results:**

Eight factors were extracted by exploratory factor analysis with a cumulative variance explanation rate was 79.45%. Confirmatory factor analysis supports the hypothesis of an 8-factor model (χ^2^/*df* = 1.846, CFI = 0.939, TLI = 0.929, SRMR = 0.050, RMSEA = 0.061), and the 8 dimensions are Endurance, Flexibility and Coordination, Body Fat, Appearance, Global Physical, Health, Sport, and Regular Exercise. The average variance extracted from each factor of the Chinese version of the PSDQ-S convergent validity index was greater than 0.50, and the composite reliability was greater than 0.60. The gender invariance hypothesis was established.

**Conclusion:**

The revised Chinese version of the PSDQ-S has good validity, reliability, and gender equivalence, encompassing eight sub-dimensions that can assess the independent yet interrelated latent constructs within the physical self-concept of Chinese middle school students.

## 1 Introduction

The Physical Self (PS) constitutes a dynamic, multidimensional construct encompassing an individual’s cognitive appraisal and affective evaluation of bodily attributes, including physical competence, appearance, health perceptions, and body composition. Emerging as the foundational component of self-concept development (Tang, 2015), this psychophysical entity evolves across the lifespan through complex interactions between biological maturation, sociocultural influences, and experiential learning (Harter, 2012). Contemporary research emphasizes three critical aspects of PS development: (1) sociocultural specificity in body-related schemas (Markus and Kitayama, 1991; Harter, 2012) heightened malleability during adolescence due to pubertal changes and identity formation (Eccles and Roeser, 2011), and (Markus and Kitayama, 1991) its hierarchical organization within global self-concept (Shavelson et al., 1976; Sonstroem, 1978).

Sociocultural research reveals significant cross-cultural variance in PS manifestation. While Western cultures prioritize individual physical competence (Marsh, 1987), Eastern collectivist societies emphasize body propriety and social harmony in physical presentation (Wang et al., 2018). These cultural scripts interact with developmental milestones—adolescents in China demonstrate heightened sensitivity to peer evaluation of physical appearance compared to Western counterparts (Shen et al., 2022), creating unique pathways for PS development. For instance, both male and female students experience body image concerns, but the nature of these concerns differs. Females tend to overestimate their body size and aspire to be thinner, while males show a mix of preferences for slimmer or fuller figures (Song et al., 2023).

Adolescent development, driven by pubertal transformations including biological changes and sexual maturation, critically shapes Physical Self-Concept (PSC) and identity formation through direct neuroendocrine impacts on affective processes, coinciding with emerging gender disparities in depression onset (Derose and Brooks-Gunn, 2008). Additionally, the physical changes of puberty prompt adolescents to reassess their childhood identities and integrate these with their evolving self-conceptions, values, and future aspirations. This process is crucial for achieving a coherent sense of self and personal autonomy (Jones et al., 2014).

The PS-behavior linkage operates through self-regulatory mechanisms. Positive changes in how individuals view their physical selves can reinforce continued exercise. Improvements in physical self-concept, such as body satisfaction and self-perception of fitness, can mediate the relationship between exercise participation and adherence (Annesi et al., 2011). Conversely, negative PS (particularly appearance) predicts social physique anxiety, dietary restraint, and eating disorders (Crocker et al., 2006; Williams and Levinson, 2020). Within educational settings, self-perception regulation demonstrates a significant mediating effect, accounting for approximately 40% of the variance in key outcomes associated with physical education engagement, including general self-esteem and health-related quality of life (HRQoL) (Standage and Gillison, 2007; Standage and Gillison, 2007). Therefore, an accurate understanding of physical self-concept is critically important for the effective implementation of China’s 2018 and 2022 editions of the Physical Education and Health Curriculum Standards.

The measurement of physical self-concept has evolved through distinct methodological phases. Following Shavelson’s foundational multidimensional model distinguishing physical ability and appearance (Shavelson et al., 1976), Sonstroem’s PEAS framework (Physical Estimation and Attraction Scales, PEAS) (Sonstroem, 1978) established the behavioral linkage between PS and sports participation. Subsequent scholars including Harter (1978), Fox and Corbin (1989), Marsh (1987), Marsh et al. (1994), Marsh et al. (2010) systematically refined the PS dimensional structure, culminating in Marsh’s 70-item Physical Self-Description Questionnaire (PSDQ). To address practical constraints, Marsh et al. (2010) developed the 40-item PSDQ-Short (PSDQ-S) through rigorous psychometric optimization, demonstrating cross-population stability (Cronbach’s α > 0.77) across Australian, Spanish, and Brazilian cohorts (Tomas et al., 2014; Dolenc, 2016; Vaz Junior et al., 2020). PSDQ-S’s scientific robustness stems from its dual validation approach: (1) hierarchical confirmatory factor analysis confirming 11 theoretically aligned dimensions, and (2) multi-group invariance testing establishing measurement equivalence across genders and ethnicities. Its universal applicability is evidenced by successful adaptations in multiple countries, maintaining structural validity (CFI > 0.92) despite cultural variations (Braun et al., 2018; Haapea et al., 2018; Brown and Bonsaksen, 2019; Rubeli et al., 2020).

However, Wang et al. (2015) Chinese adaptation revealed critical limitations. While achieving discriminant validity (d > 0.80 among three activity groups), 8/11 dimensions showed inadequate convergent validity (AVE < 0.5), particularly in culturally sensitive domains like Global Self-esteem (AVE = 0.31) and Health (AVE = 0.38). This aligns with cross-cultural psychometric critiques suggesting direct translation often fails to capture collectivist constructs of bodily propriety and social harmony (Gao et al., 2024).

These findings highlight both the feasibility and necessity of culturally informed PSDQ-S adaptation for Chinese adolescents. Middle school students in China exhibit unique PS developmental trajectories shaped by Confucian body ethics and exam-oriented educational pressures (Gao et al., 2024). We therefore propose a three-phase transcultural revision: (1) cognitive interviews to evaluate item semantic equivalence, (2) exploratory structural equation modeling to identify culture-specific factor loadings, and (3) horizontal validation against Vigorous-intensity Physical Activity (VPA). This approach balances PSDQ-S’s universal scientific framework with necessary cultural calibration.

## 2 Materials and methods

### 2.1 Participants

A convenience sampling method was employed, initially reaching 1,632 participants, with 1,584 valid questionnaires retained (valid response rate: 97.06%). The final sample consisted of 1,584 middle school students (775 males, 809 females; age range 12–18 years, *M* = 15.05, SD = 1.99) recruited from four districts in Shanghai, including both urban districts (Jing’an, Changning) and rural districts (Songjiang, Chongming) between November 2019 and March 2020. Participants were divided into two subgroups via systematic coding: odd-numbered IDs (n1 = 792) for exploratory analyses and even-numbered IDs (n2 = 792) for confirmatory analyses. Demographic distributions by grade were:

Grade 7: 275 students (150 male, 125 females, *M* = 12.22, SD = 0.33, male: female ratio = 1.2:1)

Grade 8: 332 students (170 male, 162 females, *M* = 13.09, SD = 0.29, male: female ratio = 1.05:1)

Grade 11: 977 students (455 male, 522 females, *M* = 17.11, SD = 0.34, male: female ratio = 0.87:1)

Ethical approval was obtained from East China Normal University (HR 562-2019), with informed consent secured from all participants.

### 2.2 Measures

#### 2.2.1 Chinese PSDQ-S adaptation

The 40-item PSDQ-S (Marsh et al., 2010) was adapted from the Chinese PSDQ validated in 2002 (Yang, 2002). Retaining the original 11 dimensions, the scale employed a 6-point Likert format (1 = completely disagree to 6 = completely agree), with 10 reverse-scored items (e.g., Item 6: “I often feel unhealthy”). The dimensions and their respective items were as follows:

Health (HE) dimension included five items (items 6, 15, 25, 32, and 39);

Coordination (CO) dimension included five items (items 1, 7, 16, 19, and 26);

Activity (AC) dimension included four items (items 8, 20, 27, and 33);

Body Fat (BF) dimension included three items (items 9, 17, and 21);

Sport (SP) dimension included three items (items 10, 22, and 34);

Global Physical (GP) dimension included three items (items 11, 23, and 35);

Appearance (AP) dimension included three items (items 12, 18, and 28);

Strength (ST) dimension included three items (items 2, 13, and 29);

Flexibility (FL) dimension included three items (items 3, 14, and 36);

Endurance (EN) dimension included three items (items 4, 30, and 37);

Global Esteem (ES) dimension included three items (items 5, 24, 31, 38, and 40).

The data was processed, screened, and handled by one professional staff member.

### 2.2.2 The international physical activity questionnaire

The International Physical Activity Questionnaire (IPAQ) were selected to measure physical activity and sedentary time of participants over the past week. Respondents were required to recall and report the number of days they engaged in Vigorous Physical Activity (VPA) during the past 7 days, as well as the duration (in hours or minutes) of such activity on those specific days. In addition, the average daily sedentary time from Monday to Friday was also reported.

### 2.3 Procedure

#### 2.3.1 Data collection

Eight physical education teachers from participating schools received standardized training on administering the online questionnaire via Questionnaire Star. Students completed the survey during supervised class sessions to ensure protocol adherence.

#### 2.3.2 Data screening

A trained researcher performed quality control using these criteria: (1) removal of duplicate IP submissions, (2) exclusion of responses with < 90% completion in 10 min, and (3) elimination of patterned responses (e.g., straight-line answers).

### 2.4 Data analysis

Using SPSS 24.0 and Mplus 8.3, we conducted an exploratory factor analysis (EFA) on the n1 data to examine items and dimensions; similarly, we conducted a confirmatory factor analysis (CFA) on the n2 data. We used EFA, CFA, Average Variance Extracted (AVE), and composite reliability to evaluate the validity and reliability of the questionnaire.

In the item analysis and Critical Ratio (CR) discrimination, we calculated the CR to identify items with discriminative power. The CR value was derived from an independent samples *t*-test between the high-score group (top 27%) and the low-score group (bottom 27%). If the CR value was significantly greater than 3 (*p* < 0.05) (Nunnally and Bernstein, 1994), it indicated that the item effectively differentiated self-perception levels across different groups. EFA was used to explore the latent factor structure of the scale and was suitable for dimensional validation when revising cross-cultural scales. The KMO value (>0.80) and Bartlett’s test of sphericity (*p* < 0.001) ensured data suitability for factor analysis. Factor loadings (>0.50) and cumulative variance explained (>60%) were used to retain valid items. The scree plot inflection point, combined with eigenvalues (>1), was used to determine the number of factors. In the first step, the EFA was fixed at 11 dimensions, and the model reported the extracted 11-factor parameters, which were then combined with a scree plot for analysis. In this study, the factor structure was optimized using maximum likelihood estimation and oblique rotation (Promax), with items having loadings below 0.5 removed.Multiple fit indices provided by Mplus 8.3 were examined to evaluate the adequacy of the models. The Comparative Fit Index (CFI) is an incremental fit index that compares the fit of the proposed model to the fit of a null model. The Tucker-Lewis Index (TLI) is another incremental fit index that also takes into account the complexity of the model. It ranges from 0 to 1, with values greater than 0.90 generally indicating an acceptable fit. The Standardized Root Mean Square Residual (SRMR) is an absolute fit index that measures the standardized difference between the observed and predicted covariance matrices. An SRMR value less than 0.08 generally indicates an acceptable fit, suggesting that the model fits the data reasonably well. The Root Mean Square Error of Approximation (RMSEA) is an absolute fit index that measures the discrepancy between the sample covariance matrix and the model-implied covariance matrix, adjusted for model complexity. An RMSEA value less than 0.08 generally indicates an acceptable fit, suggesting that the model approximates the data reasonably well. Additionally, standardized factor loadings needed to be significant (*p* < 0.05), meaning that the relationship between the observed variable and the latent factor is unlikely to be due to chance. The Akaike Information Criterion (AIC) and Bayesian Information Criterion (BIC) are information criteria used to compare the fit of different models while penalizing for model complexity. Models with lower AIC and BIC values are considered to have a better fit, as they achieve a good balance between model fit and simplicity.

After employing EFA to establish the relationship between observed indicators and latent variables, CFA was subsequently utilized to further examine the fit of the pre-specified factor model and validate the robustness of EFA results. To identify the constructed model, the loading of the first indicator for each factor was fixed at 1 by default, and it was assumed that the factors were correlated. The variances of the factors, residual variances of the observed items, and item intercepts were estimated freely, and the residuals of the items were specified as uncorrelated. A first-order CFA model (CFA_I_) was constructed. The AVE focuses on the average amount of variance captured by a latent variable from its indicators, reflecting the scale’s convergent validity. Higher AVE values indicate stronger convergent validity, with values greater than 0.50 generally considered acceptable. Regarding reliability, Cronbach’s α is calculated based on the average covariance among all items, which may not accurately reflect the scale’s reliability when items have different factor loadings. Composite reliability considers both factor loadings and the error variances of each item, providing a more accurate measure for assessing scale reliability during revision (e.g., when items are deleted). Composite reliability was assessed to evaluate internal consistency, with a standard higher than 0.60. We used composite reliability and AVE jointly to assess both reliability and validity in this study. This approach provides a more comprehensive and accurate assessment of the scale’s quality, particularly in structural equation modeling (SEM).

Measurement invariance across gender was examined through multi-group confirmatory factor analysis (MGCFA) using a hierarchical constraint approach. The analysis sequentially tested three levels of invariance: (a) form equivalence (unconstrained model with identical factor structure across groups), (b) loading equivalence, (c) scalar equivalence, and (d) error equivalence. Robust maximum likelihood estimation (MLR) was applied to address non-normality in the data. Invariance was evaluated using differential fit indices between nested models, with thresholds set as follows: ΔCFI ≤ 0.010, ΔRMSEA ≤ 0.015, and ΔSRMR ≤ 0.030 for metric invariance; ΔSRMR ≤ 0.010 for scalar invariance; and ΔCFI ≤ 0.010, ΔRMSEA ≤ 0.015 for strict invariance (Cheung and Rensvold, 2002). When these criteria were met, the higher-level invariance was deemed tenable, permitting valid cross-group comparisons.

After determining the cluster profiles, we used multivariate analysis of variance (MANOVA) within the general linear model framework to assess differences in VPA and sedentary time across the groups. The significance threshold was set at *p* < 0.05. This analysis helped to validate the construct validity of the cluster solution.

## 3 Research results

The mean scores, standard deviations, skewness, kurtosis, and the CR values for the PDSQ-S items were shown in Table 1. Except for the eighth item, the CR values of all the other 39 items were significantly larger than 3, indicating that these 39 items have good discriminant validity. Subsequent correlation analyses showed that these 39 items were significantly related to the total score (*r* = 0.399 ∼ 0.807). These items can be subjected to factor analysis.

**TABLE 1:** The items statistical table in the Chinese version of PSDQ-S.

Item	M ± SD	S_k_	K_u_	CR	*p*	*r*	Item	M ± SD	*S*	*K*	CR	*p*	*r*
1	4.61 ± 1.31	−0.83	−0.04	17.14	<0.001	0.571^***^	21	4.58 ± 1.75	−0.86	−0.70	11.92	<0.001	0.411^***^
2	4.31 ± 1.32	−0.55	−0.46	25.51	<0.001	0.664^***^	22	4.43 ± 1.20	−0.39	−0.57	31.74	<0.001	0.806^***^
3	4.76 ± 1.30	−0.94	0.13	18.95	<0.001	0.575^***^	23	4.45 ± 1.30	−0.71	−0.03	29.31	<0.001	0.777^***^
4	3.87 ± 1.51	−0.20	−0.96	21.09	<0.001	0.646^***^	24	5.23 ± 1.18	−1.75	2.60	16.12	<0.001	0.561^***^
5	4.65 ± 1.12	−0.74	0.32	21.25	<0.001	0.646^***^	25	5.21 ± 1.10	−1.50	1.85	12.67	<0.001	0.428^***^
6	4.97 ± 1.11	−1.05	0.59	12.80	<0.001	0.435^***^	26	4.37 ± 1.32	−0.48	−0.51	28.72	<0.001	0.710^***^
7	4.56 ± 1.24	−0.55	−0.57	21.33	<0.001	0.668^***^	27	4.07 ± 1.49	−0.25	−0.92	21.42	<0.001	0.647^***^
8	2.79 ± 1.35	0.46	−0.54	0.89	0.376	−0.022	28	4.03 ± 1.54	−0.35	−0.81	15.13	<0.001	0.524^***^
9	4.34 ± 1.63	−0.61	−0.88	12.85	<0.001	0.461^***^	29	4.13 ± 1.34	−0.31	−0.69	20.47	<0.001	0.637^***^
10	4.09 ± 1.37	−0.27	−0.78	27.14	<0.001	0.712^***^	30	3.80 ± 1.43	0.00	−0.96	29.03	<0.001	0.761^***^
11	4.15 ± 1.43	−0.41	−0.70	25.74	<0.001	0.710^***^	31	4.40 ± 1.22	−0.51	−0.19	26.22	<0.001	0.744^***^
12	4.29 ± 1.43	−0.54	−0.48	15.94	<0.001	0.539^***^	32	4.90 ± 1.19	−1.01	0.39	14.38	<0.001	0.457^***^
13	4.50 ± 1.26	−0.48	−0.53	25.69	<0.001	0.718^***^	33	4.07 ± 1.52	−0.30	−0.97	18.81	<0.001	0.583^***^
14	4.74 ± 1.15	−0.61	−0.43	26.27	<0.001	0.729^***^	34	4.27 ± 1.27	−0.31	−0.62	32.73	<0.001	0.803^***^
15	5.21 ± 1.09	−1.68	2.88	11.87	<0.001	0.410^***^	35	4.32 ± 1.36	−0.52	−0.49	32.85	<0.001	0.807^***^
16	4.41 ± 1.33	−0.53	−0.52	24.07	<0.001	0.672^***^	36	4.57 ± 1.22	−0.56	−0.41	29.12	<0.001	0.755^***^
17	4.13 ± 1.67	−0.39	−1.18	15.44	<0.001	0.521^***^	37	3.74 ± 1.61	−0.14	−1.12	19.94	<0.001	0.649^***^
18	3.84 ± 1.49	−0.16	−0.85	12.96	<0.001	0.486^***^	38	4.59 ± 1.26	−−0.58	−0.39	22.03	<0.001	0.676^***^
19	4.52 ± 1.20	−0.38	−0.76	31.67	<0.001	0.784^***^	39	5.28 ± 1.09	−1.80	3.07	12.08	<0.001	0.399^***^
20	4.77 ± 1.36	−0.88	−0.21	14.53	<0.001	0.487^***^	40	4.92 ± 1.25	−1.15	0.79	12.17	<0.001	0.408^***^

*p < 0.05, **p < 0.005,

***p < 0.001, the same as above.

### 3.1 Validity analysis

#### 3.1.1 Results of exploratory factor analysis

**FIGURE 1:**
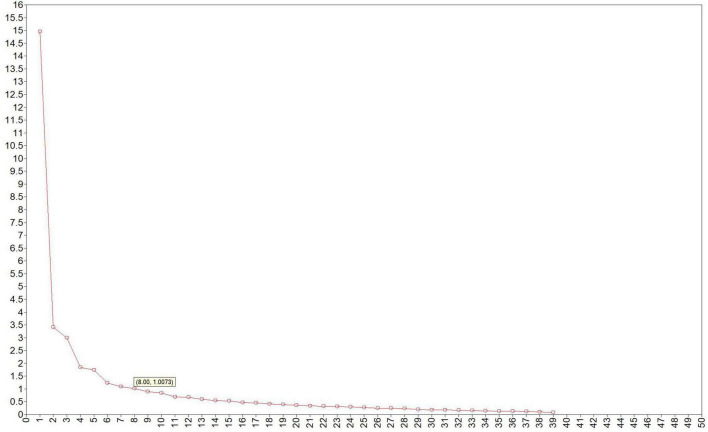
The exploratory factor gravel map in the Chinese version of PSDQ-S.

For n1, exploratory factor analysis demonstrated that the KMO value was 0.913, and Bartlett’s sphericity test χ^2^ = 6377.157, df = 435, *p* < 0.001. The skewness coefficients of items ranged between −1.80 and 0.46, and kurtosis coefficients ranged between −1.18 and 3.07, with absolute values less than 2 and 7, respectively. Thus, using maximum likelihood estimation is robust. The Mplus running result showed a clear turning point after the sixth factor (Figure 1), with eight-factor eigenvalues greater than 1. After applying maximum likelihood estimation and oblique rotation (Promax), 10 items with factor loadings below 0.5 were removed (e.g., items 2, 5, 8, 13, 19, 24, 29, 31, 38, 40). As shown in Table 2 (factor loading matrix) and Table 3 (comparative fit indices for composite robustness), the eight-factor model (EFA_8_, CFI = 0.967, TLI = 0.935, AIC = 18567.286, BIC = 19496.465, SRMR = 0.019, RMSEA = 0.058, with a 90% confidence interval [0.049, 0.068]) outperformed the six-factor model or the seven-factor model. The revised questionnaire demonstrated good structural validity. The variance explained by the eight retained common factors were 17.1812.5611.5110.53, 9.65, 8.78, 6.66 and 2.58%, respectively, with a cumulative variance explained rate of 79.45%. The factor loads of each item range from 0.50 to 0.93. The eight common factors were named as Endurance (EN), Flexibility and Coordination (FL & CO), Body Fat (BF), Appearance (AP), Global Physical (GP), Health (HE), Sport (SP), and Regular Exercise (RE), respectively.

**TABLE 2:** The factor load matrix in the Chinese version of PSDQ-S.

Factor	F1			F2							F3			F4	
Item	y4	y30	y37	y1	y3	y7	y14	y16	y26	y36	y9	y17	y21	y12	y18
Load	0.90^*^	0.68^*^	0.68^*^	0.76^*^	0.64^*^	0.56^*^	0.54^*^	0.83^*^	0.83^*^	0.66^*^	0.77^*^	0.91^*^	0.71^*^	0.87^*^	0.83^*^
**Factor**		**F5**			**F6**					**F7**			**F8**		
Item	y28	y11	y23	y35	y6	y15	y25	y32	y39	y10	y22	y34	y20	y27	y33
Load	0.93^*^	0.71^*^	0.79^*^	0.50^*^	0.71^*^	0.80^*^	0.85^*^	0.78^*^	0.89^*^	0.60^*^	0.69^*^	0.66^*^	0.60^*^	0.59^*^	0.70^*^

*p < 0.05, **p < 0.005, ***p < 0.001, the same as above.

**TABLE 3:** The models fit the index table of EFA and CFA in the Chinese version of PSDQ-S.

Model	χ^2^	*df*	TLI	CFI	AIC	BIC	SRMR	RMSEA (90% CI)
EFA_6_	1003.306	429	0.865	0.908	22229.316	23161.911	0.033	0.077 (0.071, 0.083)
EFA_7_	851.758	399	0.885	0.927	22137.768	23172.847	0.029	0.071 (0.064, 0.078)
EFA_8_	393.686	223	0.935	0.967	18567.286	19496.465	0.019	0.058 (0.049, 0.068)
CFA_I_	692.122	375	0.929	0.939	18645.161	19055.625	0.050	0.061 (0.054, 0.068)
CFA_I_	808.376	396	0.913	0.921	18719.416	19058.049	0.063	0.068 (0.061, 0.075)

#### 3.1.2 Results of confirmatory factor analysis

**FIGURE 2:**
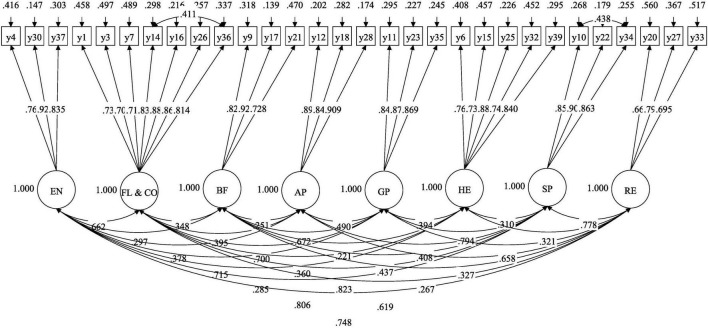
The first-order 8-factor model diagram in the Chinese version of PSDQ-S.

The first-order CFA model that was constructed achieved acceptable model fit indices (see Table 3 and Figure 2, χ^2^/df = 1.846, CFI = 0.939, TLI = 0.929, SRMR = 0.050, RMSEA = 0.061 [0.054, 0.068]). These results indicated that the factors did not include items that failed to reflect the relevant latent variables, suggesting good discriminant validity.

To ascertain whether the higher-order factors in Chinese version of the PSDQ-S for middle schoolers could effectively integrate the eight lower-order factors (EN, FL and CO, BF, AP, GP, HE, SP, and RE), a second-order CFA model (CFA_I_) was further constructed. The theoretical basis of this model assumes that the higher-order factor (e.g., “Physical Self-Concept”) integrates the common variance of the lower-dimensional factors through latent paths, while the lower-dimensional factors (e.g., “HE,” “SP,” etc.) retain their unique variance. Specifically, the higher-order factor needs to meet two core conditions: (1) theoretical rationality, that is, the lower-dimensional factors conceptually belong to the same higher-order construct; and (2) statistical feasibility, that is, the lower-dimensional factors need to show moderate or higher correlations, indicating that they share sufficient variance to converge on the higher-order factor (Honglei et al., 2014).

The model fit results showed that although the second-order model met the basic fitting standards (Table 3, χ^2^/df = 2.041, CFI = 0.921, TLI = 0.913, SRMR = 0.063, RMSEA = 0.068 [0.061, 0.075]), its fit was significantly worse than that of the first-order model: the critical value of chi-square for the degrees of freedom difference Δdf = 21 was 32.67, while the actual chi-square difference value reached 116.254 (*p* < 0.001); at the same time, the TLI and CFI of the second-order model decreased compared to the first-order model, and the AIC and BIC increased, markedly deteriorating the model’s fit.

#### 3.1.3 Results of convergence validity test

The AVE for each factor was calculated using the formula $\frac{\left(\sum \lambda^{2}\right)}{N}$. The results showed that the AVEs for the eight sub-dimensions of EN, FL & CO, BF, AP, GP, HE, SP, and RE were 0.70, 0.64, 0.70, 0.78, 0.75, 0.63, 0.76, and 0.52, respectively. All of these values were greater than 0.5, indicating that latent variables can effectively reflect the variation of their corresponding indicators (rather than the variation caused by measurement errors).

### 3.2 The results of composite reliability test results

The composite reliability of each factor was computed using the formula $\frac{{\left(\sum \lambda \right)}^{2}}{{\left(\sum \lambda \right)}^{2}+\sum \varepsilon }$. The findings revealed that the composite reliabilities for the eight sub-dimensions—EN, FL and CO, BF, AP, GP, HE, SP, and RE—were 0.88, 0.92, 0.87, 0.91, 0.90, 0.90, 0.91, and 0.76, respectively, all of which exceeded 0.60, suggesting that the model possesses a commendable inherent quality.

### 3.3 The results of measurement invariance

The results of measurement invariance showed metric invariance (see Table 4, Model B, ΔCFI = −0.001, ΔRMSEA = 0.007, and ΔSRMR = 0.001), scalar invariance (Model C, ΔCFI = −0.005, ΔRMSEA = −0.005, andΔSRMR = 0.006) and strict invariance (Model D,ΔCFI = −0.005, ΔRMSEA = 0.000).

**TABLE 4:** The gender invariance test table in the Chinese version of PSDQ-S.

Model	χ^2^	*df*	TLI	CFI	AIC	BIC	SRMR	RMSEA (90% CI)	ΔTLI	ΔCFI
A	1061.942	749	0.923	0.933	18626.083	19450.432	0.060	0.061 [0.052, 0.069]	_	_
B	1092.337	771	0.923	0.932	18610.855	19359.952	0.061	0.068 [0.052, 0.069]	0.000	−0.001
C	1115.438	771	0.917	0.927	18637.287	19386.384	0.067	0.063 [0.055, 0.071]	−0.006	−0.005
D	1165.530	801	0.916	0.922	18666.307	19312.788	0.068	0.063 [0.055, 0.071]	−0.001	−0.005

A, represents form equivalence; B, represents loading equivalence; C, represents scalar equivalence; D, represents error equivalence.

### 3.4 The assessment of physical activity by the revised PSDQ-S for Chinese middle school students

**FIGURE 3:**
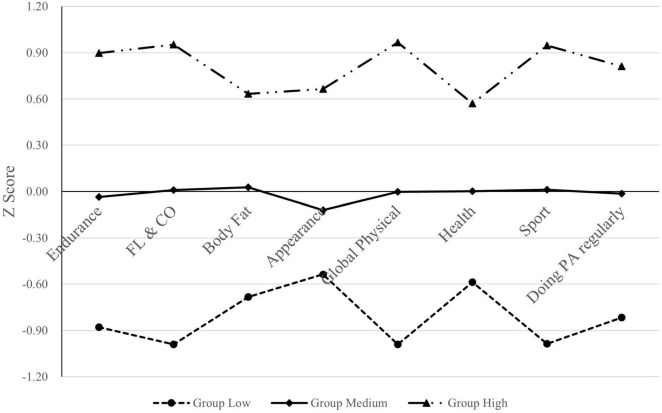
Z score graph of the revised PSDQ—S among three groups of participants.

Figure 3 illustrates the profiles of the three distinct groups. A threshold of Z-scores ± 0.50 was applied to classify self-reported scores across the eight dimensions of the revised PSDQ-S as “relatively high” or “relatively low.” Low Group (30.8% of participants, *N* = 488): Comprising 39.1% males and 60.9% females, this group had all PSDQ-S Z-scores below −0.50, indicating consistently lower self-perceptions across all dimensions. Medium Group (37.5% of participants, *N* = 594): With 46.0% males and 54.0% females, this group demonstrated Z-scores clustered near the mean (approximating 0), reflecting moderate self-evaluations. High Group (31.7% of participants, *N* = 502): Characterized by elevated PSDQ-S scores (Comprising 50.4% males and 49.6% females), this group displayed near-maximal self-perception metrics. Notably, the High and Low groups diverged by nearly 2 standard deviations on the FL & CO, GP, and SP subscales, underscoring significant intergroup contrasts in these domains.

**FIGURE 4:**
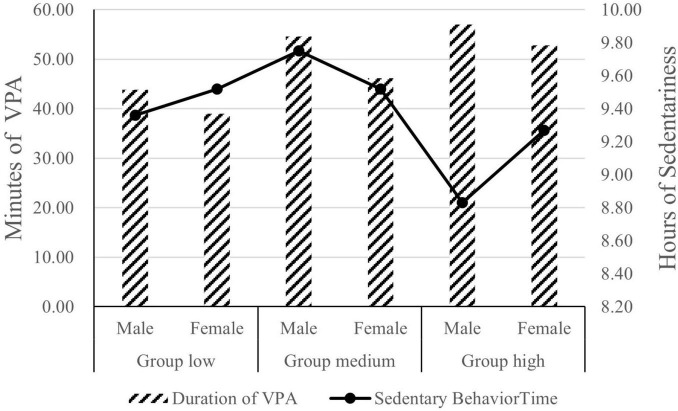
A comparison chart of vigorous physical activity and sedentary time among three groups of participants.

Statistical analysis using MANOVA with three levels and gender as independent variables, and VPA duration as the dependent variable revealed significant main effects for both factors (Figure 4). Group effect: A progressive increase in VPA duration was observed across the low, medium, and high groups [*F*(2, 6471.86) = 11.81, *p* < 0.001, partial η^2^ = 0.05]. Gender effect: Males exhibited significantly longer VPA durations than females [*F*(1, 3575.62) = 6.53, *p* < 0.05, partial η^2^ = 0.01]. Post hoc comparisons using Bonferroni further demonstrated: Group high surpassed the group low by 13.32 min (*p* < 0.001). Group medium exceeded group low by 8.85 min (*p* < 0.01). The difference between group high and group medium (4.47 min) was non-significant (*p* > 0.05). Males maintained a 5.63-min advantage over females (*p* < 0.05). Contrastingly, when sedentary time served as the dependent variable: No significant main effects emerged for group [*F* (2, 14.06) = 2.94, *p* > 0.05, partial η^2^ = 0.01] or gender [*F* (1, 1.69) = 0.35, *p* > 0.05, partial η^2^ = 0.001].

## 4 Discussion

Physical self-concept is a critical indicator in the realm of health research within sports psychology due to its significant impact on both mental and physical health outcomes. Kleinert and Tietjens’ Study showed that where improvements in PSC were associated with enhanced physical wellbeing in rehabilitation settings (Kleinert and Tietjens, 2014). A cross-sectional field survey in Indonesia found that student-athletes’ PSC mediated the relationship between their physical health and their physical activity (Gultom et al., 2021). Zhang and Chen (2005) contend that assessing the physical self has been a persistent challenge in sports psychology. This study, following a thorough analysis of PS measurement instruments, centers on the PSDQ-S, a tool extensively utilized in academic settings but exhibiting poor convergent validity when employed in China. The findings indicate that the revised Chinese version of the PSDQ-S boasts commendable reliability and validity, with eight dimensions—EN, FL and CO, BF, AP, GP, HE, SP, and RE—aptly capturing the nuances of Chinese middle school students’ Physical self-concept. This addresses the paucity of localized research on the PSDQ-S in China.

### 4.1 Characteristics of the Chinese version of PSDQ-S among middle school students

The results reveal notable similarities and discrepancies between the Chinese and English versions of the PSDQ-S. Firstly, The revised Chinese version of the PSDQ-S retained 30 items from the original English 40-item scale, with 10 items excluded based on psychometric evaluation. Due to the fact that Chinese middle school students spent less time on intense exercise than their foreign peers during the COVID-19 period, the entry “I often participate in exercises/activities that make me breathe heavily” (item 8) was poorly differentiated, so it was not included in the first round of exploratory factor analysis. Its mean score (2.79 ± 1.35) implies that sustained moderate-to-high intensity exercise may represent the “ceiling” of daily physical activity for middle school students. This discrepancy may be attributed to the divergent academic pressures faced by Chinese middle school students versus their international peers, resulting in less time allotted for self-directed exercise. Moreover, this suggests that school physical education should intensify and refine activities that foster cardiovascular health (Dunton et al., 2006), thereby laying a physiological foundation for the “improving physical fitness” philosophy within high-quality school physical education in the contemporary era. Following the removal of item 8, the original “physical activity” factor was condensed to three items (items 20, 27, and 33), reflecting the scenario where the subjects “exercise at least three times a week or nearly every day.” This factor was subsequently renamed the “Regular Exercise” factor.

Second, the Chinese adaptation of the PSDQ-S exhibits a streamlined structure comprising eight subscales, a reduction from the original English version’s 11 dimensions. To rigorously validate this refined framework, EFA and CFA were conducted on independent datasets. Psychometric evaluations revealed superior construct validity in the Chinese version: all eight subscales achieved average variance extracted (AVE) values > 0.50, compared to only 3/11 subscales meeting this threshold in Wang et al. ‘s original validation study. Furthermore, composite reliability values for the Chinese subscales ranged from 0.76 to 0.92, demonstrating marked improvement over the original version where only 5/11 dimensions exceeded the 0.70 reliability benchmark. During the EFA, the original coordination and flexibility dimensions were merged in the six, seven, and eight-factor models. From the perspective of cross-loading, the six (EFA_6_) and seven-factor models (EFA_7_) included the “Regular Exercise” (item 27) and “Coordination” (item 19) factor items within the “Sport” dimension. Therefore, the eight-factor model, which showed high fit and no cross-loading, was chosen, and the merged factors were renamed the “Flexibility and Coordination” dimension. This is consistent with the research results of Zeng and Gong (2006) on coordination. Middle school students consider ball sense and ball feeling during technical practice, cooperation during tactical practice, interpersonal relationships and communication during psychological practice, and adaptation in the competition environment as coordination. Additionally, the eight-factor model does not include the original “strength” and “global self-esteem” dimensions. The failure of the original both dimensions in the Chinese sample may be attributed to three perspectives: (1) Discrepancies in physical education emphasis. The “diversified-specialized” physical education curriculum implemented in Shanghai since 2012 has resulted in a relatively low proportion of strength training, leading to adolescents’ vague cognition of the “strength” dimension. (2) Insufficient cultural adaptation of measurement tools. The original scale’s strength-related items (e.g., “I could do well in a test of strength”) are disconnected from Chinese students’ actual performance in national physical fitness tests (e.g., pull-ups) (Dong et al., 2024); 3) Influence of social value orientation, Chinese adolescents tend to prioritize “health” and “body fat” —dimensions that align with societal aesthetic standards, while the explicit expressions of “global self-esteem” (e.g., “I have a lot to be proud of”) conflict with the cultural norm of modesty.

Thirdly, the Chinese version of the eight-factor PSDQ-S demonstrates high convergent validity, with the corresponding eight factors aligning with those in the eleven-factor English version of the PSDQ-S. This contrasts with the findings of Wang et al., who identified convergent validity for only three factors. The discrepancy is primarily attributable to the similar age range (12-18 years) and consistent psychological structure between this study and the English version of the PSDQ-S. Wang’s study included participants younger than 12 years old, who exhibit significant psychological differences from children aged 12 and older. Furthermore, this study increased the sample size (from the original cohort of 744 participants to 1,584) and enhanced the compatibility of the PSDQ-S with the self-description constructs of Chinese middle school students through dimensionality reduction. It is important to note that the Sport dimension of the PSDQ-S reflects the participants’ proficiency in sports and athletic skills, distinguishing it from the basic motor skills assessed in the “Endurance” and “Flexibility and Coordination” dimensions. Similarly, the original “Global Physical” dimension, represents a positive evaluation of one’s physical fitness. The results have indicated that the subscales are measuring independent but correlated latent constructs, lending support to their discriminant validity. This can be taken as evidence of multidimensionality within the physical self-domain. It is a hypothesis that has not previously been systematically tested.

Our analysis further revealed that the revised second-order model exhibited inferior fit indices compared to the first-order model. This phenomenon may stem from two plausible explanations: (1) Independence of lower-order factors, where specific factors (e.g., BF and AP) demonstrated insufficient inter-factor correlations (*r* = 0.25). This suggests that Chinese adolescents cognitively differentiate between “body fat perception” and “facial attractiveness” as distinct constructs, thereby failing to converge effectively under a higher-order factor; (2) Cultural specificity, as the Western theoretical framework’s higher-order structure (e.g., “independent view of self”) may diverge from the psychological constructs of Chinese adolescents (e.g., “interdependent view of self”), resulting in incomplete theoretical coverage of the higher-order factor (3).

### 4.2 Prospective applications of the Chinese adaptation of PSDQ-S among middle school students

The assessment of participants’ PA based on grouping by high and low scores on the revised Chinese version of PSDQ-S demonstrated that this instrument exhibits relatively high accuracy in evaluating VPA among Chinese middle school students. From the perspective of application prospects, as China advances layer by layer in its socialist modernization and economic, political, cultural, social, and ecological construction, the focus on high-quality school sports development has shifted to providing students with more substantial benefits in school sports, in addition to emphasizing student pass rates for physical fitness tests and quality evaluation. The Chinese version of the PSDQ-S will serve as a measurement tool for assessing the sense of gain that middle school students derive from school sports education.

From the perspective of building a sports power, the “Regular Exercise” dimension characterizes the healthy behavior of middle school students’ consistent participation in sports, which, to some extent, reflects the core sports literacy of young people and can effectively predict their level of engagement in mass sports as they enter society.

From the perspective of a Healthy China, the “Health and Body Fat” dimensions describe the body’s ability to resist flu and viruses, illness, medical treatment, and recovery conditions, providing decision-making references for solidifying the guiding ideology of “Health First” in school sports and implementing the Healthy China 2030 Plan.

From the perspective of realizing the great rejuvenation of the Chinese nation and advancing the cause of the Party, the “EN, FL and CO, SP” dimensions delineate the physical enhancement requirements for the builders and successors of the nation under the framework of prosperous, democratic, civilized, harmonious, and beautiful socialism.

At the same time, this study’s participants are the same age as those in the PSDQ-S (sample one) and are limited to “middle school students” to avoid the issue of adverse convergent validity when incorrectly applying Chinese version of the PSDQ-S to children under 12 years old.

The COVID-19 pandemic has heightened the complexity of conducting surveys, resulting in an extended research timelines. The evaluation of sedentary behavior through stratification based on revised PSDQ-S scores further confirmed that Chinese middle school students generally exhibited prolonged sedentary time during the pandemic. Methodological considerations should acknowledge that the data collection period (November 2019 to March 2020) coincided with the initial outbreak of COVID-19 in China. Three pandemic-related factors may have influenced the results: First, intermittent school closures could have affected sample representativeness, particularly through the suspension of standardized physical assessments in some institutions. Second, necessary adaptations in administration protocols (e.g., transitioning from in-person to online formats) may have introduced measurement variance in self-evaluation items. Third, restricted physical activity opportunities during lockdown periods likely modified participants’ baseline exercise behaviors and subsequent body perceptions. While these contextual factors do not fundamentally challenge the study’s validity, they underscore the importance of documenting environmental contingencies in future cross-cultural physical self-concept research. As an instrument for measuring adolescent mental health status, future research under normalized circumstances should prioritize the following: (1) deeper exploration of psychological constructs in the “strength” and “global self-esteem” dimensions among Chinese adolescents; (2) examination of the concurrent validity between the Chinese PSDQ-S and health indicators (Dunton et al., 2006), physical activity levels (including sedentary duration), and motor proficiency.

The study’s limitations manifest in three primary aspects: geographical constraints, instrument generalizability, and unexamined demographic variables. Specifically, the exclusive focus on Shanghai may inadequately represent adolescents’ physical self-perceptions and expressions across diverse cultural contexts. Although the questionnaire has undergone validity and reliability assessments, its applicability to non-Han ethnic linguistic contexts in China requires further verification. While gender measurement invariance has been established, the potential influence of socioeconomic status remains unaddressed. Future research should aim to enhance methodological rigor, external validity, and survey design through three key strategies: (1) expanding geographical sampling scope; (2) employing cross-validation or alternative approaches to confirm findings’ robustness; (3) extending generalizability across diverse populations and ethnic environments. Additionally, innovative designs should be developed to differentiate the impacts of various factors on self-reported physical self-concepts.

## 5 Conclusion and recommendations

### 5.1 Conclusion

The revised Chinese version of the Physical Self-Description Questionnaire-Short, with its validated eight sub-dimensions (Endurance, Flexibility and Coordination, Body Fat, Appearance, Global Physical, Health, Sport, and Regular Exercise) and robust psychometric properties, holds significant potential for practical applications in Chinese physical education and adolescent health promotion. Below are evidence-based prospects for its implementation: (1) Personalized Physical Education Programming. The 30-item scale’s multidimensional structure enables educators to identify students’ strengths and weaknesses across distinct physical self-concept domains. For instance, low scores in “Endurance” or “Flexibility and Coordination” could inform tailored exercise regimens, while concerns in “Body Fat” or “Appearance” may signal the need for integrated health education to address body image issues. Such granular insights align with China’s “Healthy China 2030” initiative, which emphasizes individualized health management. (2) Objective Monitoring of Curriculum Effectiveness. As a standardized tool, the PSDQ-S could serve as a pre- and post-intervention metric to evaluate the impact of school-based physical activity programs. For example, improvements in “Sport” or “Regular Exercise” scores after implementing mandatory daily exercise policies would provide empirical evidence of policy efficacy, aiding evidence-driven adjustments to national physical education guidelines. (3) Early Identification of At-Risk Adolescents. The “Health” and “Global Physical” subscales may function as early warning systems for students with declining physical self-perception—a known correlate of sedentary behavior and mental health risks. Integrating the PSDQ-S into routine school health screenings could facilitate timely psychosocial interventions, particularly in post-pandemic contexts where prolonged sedentary lifestyles persist. (4) Cross-Cultural Adaptation and Policy Standardization While validated in Chinese middle schoolers, the scale’s applicability to ethnic minority populations (e.g., Tibetan or Uyghur students) remains untested. Systematic cross-regional studies using this instrument could inform the development of culturally sensitive physical education frameworks, addressing disparities in China’s diverse educational landscapes. (5) Linking Physical Self-Concept to Academic Outcomes. Future studies could leverage the PSDQ-S to investigate hypothesized relationships between domain-specific self-concepts (e.g., “Sport” competence) and academic performance, potentially reinforcing physical education’s role in holistic student development. This aligns with global trends advocating for physically active learning models. (6) Teacher Training and Pedagogical Innovation. Incorporating PSDQ-S data into teacher professional development programs could enhance educators’ ability to design inclusive curricula that address gender differences (e.g., boys’ vs. girls’ perceptions of “Endurance”) and socioeconomic barriers to physical activity participation.

### 5.2 Methodological recommendations

To maximize translational value, large-scale longitudinal studies should establish normative PSDQ-S benchmarks across China’s geographic and socioeconomic strata. Concurrent validation with objective measures (e.g., accelerometry for “Regular Exercise,” skinfold tests for “Body Fat”) would strengthen its utility in bridging psychological constructs with physiological health outcomes. Furthermore, integrating the scale into digital health platforms could enable real-time monitoring of population-level trends, informing dynamic policy adjustments in China’s evolving educational ecosystem. By anchoring physical education practices in empirically derived self-concept profiles, the PSDQ-S offers a scientifically grounded pathway to optimize adolescent health strategies while addressing the unique challenges of China’s rapidly urbanizing and technologically immersive learning environments.

## Data Availability

The raw data supporting the conclusions of this article will be made available by the authors, without undue reservation.

## References

[B1] Annesi J. Unruh J. Marti C. Gorjala S. Tennant G. (2011). Effects of the coach approach intervention on adherence to exercise in obese women: Assessing mediation of social cognitive theory factors. *Res. Q. Exerc. Sport*. 82 99–108. 10.1080/02701367.2011.10599726 21462690

[B2] Braun A. Martin T. Alfermann D. Michel S. (2018). Analysis of the reliability and validity of the short version of the physical self-description questionnaire (PSDQ-S) for persons of early and late adulthood. *Z. Sportpsychol*. 25 115–127. 10.1026/1612-5010/a000236

[B3] Brown T. Bonsaksen T. (2019). An examination of the structural validity of the physical self-description questionnaire-short form (PSDQ-S) using the rasch measurement model. *Cogent. Educ.* 6:1571146. 10.1080/2331186X.2019.1571146

[B4] Cheung G. Rensvold R. (2002). Evaluating goodness-of-fit indexes for testing measurement invariance. *Struct. Equ. Model*. 9 233–255. 10.1207/S15328007SEM0902_5

[B5] Crocker P. Sabiston C. Kowalski K. Mcdonough M. Kowalski N. (2006). Longitudinal assessment of the relationship between physical self-concept and health-related behavior and emotion in adolescent girls. *J. Appl. Sport Psychol*. 18 185–200. 10.1080/10413200600830257

[B6] Derose L. Brooks-Gunn J. (2008). *Pubertal Development in Early Adolescence: Implications for Affective Processes.* Cambridge, MA: Cambridge University Press, 56–73. 10.1017/CBO9780511551963.004

[B7] Dolenc P. (2016). The short form of the physical self-description questionnaire: Validation study among Slovenian elementary and high school students. *J. Psychol. Educ. Res*. 24 58–74.

[B8] Dong G. Du P. Cui Y. Huang A. (2024). The practical problems and resolution paths of China’s physical education entrance examination reform for senior high schools under the background of the implementation of the new curriculum standard. *J. Phys. Educ*. 31 113–119. 10.16237/j.cnki.cn44-1404/g8.2024.04.009

[B9] Dunton G. Schneider M. Graham D. Cooper D. (2006). Physical activity, fitness, and physical self-concept in adolescent females. *Pediatr. Exerc. Sci*. 18 240–251. 10.1123/pes.18.2.240

[B10] Eccles J. Roeser R. (2011). Schools as developmental contexts during adolescence. *J. Res. Adolesc.* 21 225–241. 10.1111/j.1532-7795.2010.00725.x

[B11] Fox K. Corbin C. (1989). The physical self-perception profile - development and preliminary validation. *J. Sport Exerc. Psychol*. 11 408–430. 10.1123/jsep.11.4.408

[B12] Gao M. Che W. Qi S. (2024). The construction of a physical literacy assessment index system for junior high school students: A Chinese study. *Sci. Rep*. 14:23938. 10.1038/s41598-024-74698-6 39397095 PMC11471848

[B13] Gultom S. Endriani D. Harahap A. Baharuddin B. Fibriasari H. (2021). Exploring the perceptions of student-athletes leading towards physical activity and perceived performance. *Rev. Psicol. Deporte*. 30 201–215.

[B14] Haapea I. Haverinen K. Honkalampi K. Kuittinen M. Räty H. (2018). The factor structure and reliability of the short form of the Physical Self-Description Questionnaire in a Finnish adolescent athlete sample. *Int. J. Sport Exerc. Psychol*. 16 488–504. 10.1080/1612197X.2016.1266504

[B15] Harter S. (1978). Effectance motivation reconsidered: Toward a developmental model. *Hum. Dev.* 21 34–64. 10.1159/000271574

[B16] Harter S. (2012). *The Construction of the Self: Developmental and Sociocultural Foundations*, 2nd Edn. New York, NY: The Guilford Press.

[B17] Honglei G. Zhonglin W. Jie F. (2014). Bi-factor models: A new measurement perspective of multidimensional constructs. *J. Psychol. Sci*. 37 973–979.

[B18] Jones R. Dick A. Coyl-Shepherd D. Ogletree M. (2014). Antecedents of the male adolescent identity crisis: Age, grade, and physical development. *Youth Soc*. 46 443–459. 10.1177/0044118X12438904

[B19] Kleinert J. Tietjens M. (2014). [Do actual body experiences affect physical self-concept? A longitudinal study with patients with cardiac disease during sport therapy]. *Psychother. Psychosom. Med. Psychol*. 64 275–283. 10.1055/s-0033-1361156 24343312

[B20] Markus H. Kitayama S. (1991). Culture and the self - implications for cognition, emotion, and motivation. *Psychol. Rev*. 98 224–253. 10.1037/0033-295X.98.2.224

[B21] Marsh H. (1987). The hierarchical structure of self-concept and the application of hierarchical confirmatory factor-analysis. *J. Educ. Meas*. 24 17–39. 10.1111/j.1745-3984.1987.tb00259.x

[B22] Marsh H. Martin A. Jackson S. (2010). Introducing a short version of the physical self description questionnaire: New strategies, short-form evaluative criteria, and applications of factor analyses. *J. Sport Exerc. Psychol*. 32 438–482. 10.1123/jsep.32.4.438 20733208

[B23] Marsh H. Richards G. Johnson S. Roche L. Tremayne P. (1994). Physical self-description questionnaire - psychometric properties and a multitrait-multimethod analysis of relations to existing instruments. *J. Sport Exerc. Psychol*. 16 270–305. 10.1123/jsep.16.3.270

[B24] Nunnally J. Bernstein I. (1994). *Psychometric Theory*, 3rd Edn. New York, NY: McGraw-Hill.

[B25] Rubeli B. Oswald E. Conzelmann A. Schmid J. Valkanover S. Schmidt M. (2020). Promoting schoolchildren’s self-esteem in physical education: Testing the effectiveness of a five-month teacher training. *Phys. Educ. Sport Pedagogy*. 25 346–360. 10.1080/17408989.2020.1712348

[B26] Shavelson R. Hubner J. Stanton G. (1976). Self-concept: Validation of construct interpretations. *Rev. Educ. Res*. 46 407–441. 10.3102/00346543046003407 38293548

[B27] Shen J. Chen J. Tang X. Bao S. (2022). The effects of media and peers on negative body image among Chinese college students: A chained indirect influence model of appearance comparison and internalization of the thin ideal. *J. Eat. Disord*. 10:49. 10.1186/s40337-022-00575-0 35413877 PMC9006462

[B28] Song H. Cai Y. Cai Q. Luo W. Jiao X. Jiang T. (2023). Body image perception and satisfaction of junior high school students: Analysis of possible determinants. *Children* 10:1060. 10.3390/children10061060 37371291 PMC10297467

[B29] Sonstroem R. (1978). Physical estimation and attraction scales: Rationale and research. *Med. Sci. Sports* 10 97–102. 692310

[B30] Standage M. Gillison F. (2007). Students’ motivational responses toward school physical education and their relationship to general self-esteem and health-related quality of life. *Psychol. Sport Exerc*. 8 704–721. 10.1016/j.psychsport.2006.12.004

[B31] Tang Z. (2015). *New Action Theory Series: Social Psychology of Sports.* Shanghai: East China Normal University Press.

[B32] Tomas I. Marsh H. Gonzalez-Roma V. Valls V. Nagengast B. (2014). Testing measurement invariance across Spanish and English versions of the physical self-description questionnaire: An application of exploratory structural equation modeling. *J. Sport Exerc. Psychol*. 36 179–188. 10.1123/jsep.2013-0070 24686954

[B33] Vaz Junior A. Mouad M. Cavazzotto T. Serassuelo Junior H. (2020). Validation of the brazilian version of the physical self-description questionnaire - short. *Rev. Andaluza Med. Deporte*. 13 55–59. 10.33155/j.ramd.2020.02.004

[B34] Wang C. Sun Y. Liu W. Yao J. Pyun D. (2015). Latent profile analysis of the physical self-description among Chinese adolescents. *Curr. Psychol*. 34 282–293. 10.1007/s12144-014-9257-y

[B35] Wang K. Liang R. Ma Z. Chen J. Cheung E. Roalf D. (2018). Body image attitude among Chinese college students. *Psych. J*. 7 31–40. 10.1002/pchj.200 29297988

[B36] Williams B. Levinson C. (2020). Negative beliefs about the self prospectively predict eating disorder severity among undergraduate women. *Eat. Behav.* 37:101384. 10.1016/j.eatbeh.2020.101384 32320928 PMC7246166

[B37] Yang J. (2002). Introduction and revision of PSDQ. *Shandong Sports Sci. Technol.* 24 83–86. 10.14105/j.cnki.1009-9840.2002.01.038

[B38] Zeng G. Gong B. (2006). The lost and dislocation phenomenon analysis of coordination in youth football training in China. *J. Wuhan Inst. Phys. Educ.* 11, 82–84.

[B39] Zhang L. Chen L. (2005). Comparison of six physical self-measurement methods. *Sports Sci.* 1, 74–79.

