# Supplementary material for: The revised version of the Physical Self-Description Questionnaire-Short for Chinese middle school students
Source: Front Psychol. 2025 Jun 5;16:1513882. doi: 10.3389/fpsyg.2025.1513882 (PMC12179392; doi:10.3389/fpsyg.2025.1513882)
Supplement: Supplementary file 1 [file Supplementary_file_1.docx]

Supplementary Material

# S1 Physical Self-Description Questionnaire-Short, PSDQ-S

To investigate the attitudes of adolescents towards physical exercise and their physical health status, a survey and research are being conducted in Shanghai as part of the " Li De Shu Ren Humanities and Social Sciences Base (Sports)" project. The following questionnaire has six options for the degree of agreement (1 represents "Completely Disagree," 6 represents "Completely Agree," and 2-5 are intermediate levels between "Completely Disagree" and "Completely Agree"), with no right or wrong answers. Please fill it out carefully according to your true situation. This questionnaire is to be filled out anonymously and is for academic research only, and your privacy will be protected. Thank you very much for your support and assistance!

01. I feel confident when doing coordinated movements. 1 2 3 4 5 6

02. I am a physically strong person. 1 2 3 4 5 6

03. I am quite good at bending, twisting and turning my body. 1 2 3 4 5 6

04. I can run a long way without stopping. 1 2 3 4 5 6

05. Overall, most things I do turn out well. 1 2 3 4 5 6

06. I usually catch whatever illness (flu, virus, cold etc) is going around. 1 2 3 4 5 6

07. Controlling movements of my body comes easily to me. 1 2 3 4 5 6

08. I often do exercise or activities that make me breathe hard. 1 2 3 4 5 6

09. My waists too large. 1 2 3 4 5 6

10. I am good at most sports. 1 2 3 4 5 6

11. Physically, I am happy with myself. 1 2 3 4 5 6

12. I have a nice looking face. 1 2 3 4 5 6

13. I have a lot of power in my body. 1 2 3 4 5 6

14. My body is flexible. 1 2 3 4 5 6

15. I am sick so often that I cannot do all the things I want to do. 1 2 3 4 5 6

16. I am good at coordinated movements. 1 2 3 4 5 6

17. I have too much fat on my body. 1 2 3 4 5 6

18. I am better looking than most of my friends. 1 2 3 4 5 6

19. I can perform movements smoothly in most physical activities. 1 2 3 4 5 6

20. I do physically active things (e.g. jog, dance, bicycle, aerobics, gym, swim) at least three times a week. 1 2 3 4 5 6

21. I am overweight. 1 2 3 4 5 6

22. I have good sports skills. 1 2 3 4 5 6

23. Physically, I feel good about myself. 1 2 3 4 5 6

24. Overall, I am no good. 1 2 3 4 5 6

25. I get sick a lot. 1 2 3 4 5 6

26. I find my body handles coordinated movements with ease. 1 2 3 4 5 6

27. I do lots of sports, dance, gym, or other physical activities. 1 2 3 4 5 6

28. I am good looking. 1 2 3 4 5 6

29. I could do well in a test of strength. 1 2 3 4 5 6

30. I can be physically active for a long period of time without getting tired. 1 2 3 4 5 6

31. Most things I do, I do well. 1 2 3 4 5 6

32. When get sick, it takes me a long time to get better 1 2 3 4 5 6

33. I do sports, exercise, dance or other physical activities almost every day. 1 2 3 4 5 6

34. I play sports well. 1 2 3 4 5 6

35. I feel good about who am physically. 1 2 3 4 5 6

36. I think would perform well on a test measuring flexibility. 1 2 3 4 5 6

37. I am good at endurance activities like distance running, aerobics, bicycling, swimming, or cross-country, skiing. 1 2 3 4 5 6

38. Overall, I have a lot to be proud of. 1 2 3 4 5 6

39. I have to go to the doctor because ofllness more than most people my age. 1 2 3 4 5 6

40. Nothing I ever do seems to turn out right. 1 2 3 4 5 6
